# Hypoplastic Myelodysplastic Syndrome: Symptom of Methotrexate Toxicity in Rheumatoid Arthritis

**DOI:** 10.7759/cureus.40580

**Published:** 2023-06-17

**Authors:** Adil Khan, Maryem Anwar, Adila Azam, Sarah Nisar, Anees ur Rehman

**Affiliations:** 1 Internal Medicine, Khyber Medical College, Peshawar, PAK; 2 Family Medicine, NHS (National Health Service), Slough, GBR; 3 Paediatrics, Leeds Teaching Hospitals NHS (National Health Service) Trust, Leeds, GBR; 4 Medicine, Ayub Medical College, Peshawar, PAK

**Keywords:** dmard therapy, folinic acid, “pancytopenia”, rheumatoid arthritis, methotrexate-induced hypoplastic myelodysplasia

## Abstract

Methotrexate is the conventional disease-modifying anti-rheumatic drug (DMARD) which is considered the drug of choice in the treatment of rheumatoid arthritis, but its prolonged use without monitoring leads to a number of complications involving different body systems. The toxic effects of long-term methotrexate (MTX) therapy mainly involve the liver, skin, gastrointestinal tract (GIT) and bone marrow. In the bone marrow, it mainly causes suppression of normal functionality, leading to the formation of abnormal blast cells and dysplasia. In this case report, we present a male patient with symptoms of hoarseness, fatigue and abnormal bleeding all of which can be affiliated with methotrexate-induced hypoplastic myelodysplasia. As pancytopenia can be a lethal complication of MTX toxicity, it is important to monitor the therapy and dosage of methotrexate so that in case of any unforeseen development of a complication vital steps may be taken to diagnose and treat it in time. Regarding our patient, after thorough history taking and undergoing extensive hematological workup, the diagnosis of MTX-induced hypoplastic myelodysplasia was made. His symptoms improved on withholding the drug methotrexate from his active regimen and adding folinic acid and colony-stimulating factors.

## Introduction

Rheumatoid arthritis (RA) is one of the most common autoimmune disorders. It affects 0.5%-1% of the adult population worldwide [1]. Rheumatoid arthritis is a chronic, progressive, autoimmune disease that mainly involves the joints of the body, i.e., from small to large but with further disease progression and severity; there is the eventual presentation of extra-articular features as well. Therefore, therapy for RA is directed toward reducing joint inflammation and pain, maximizing joint function, and preventing joint destruction and deformity. The main goal of initial therapy is to relieve pain and decrease inflammation. The medications of choice in this regard are nonsteroidal anti-inflammatory drugs (NSAIDs), all of which are of equal potential. However, to promote remission that is by slowing or stopping the progression of joint destruction and deformity, disease-modifying anti-rheumatic drugs (DMARDs) are prescribed; among these, methotrexate (MTX) is the most common conventional DMARD used for rheumatoid arthritis [2]. Recent therapy includes Janus kinase inhibitors (JAKi) can be used as an alternative, in cases of in-effectivity of DMARDs [3].

Methotrexate in the initial stages of the treatment of patients with rheumatoid arthritis improves pain, functionality of the joints and other symptoms. It also reduces joint damage which may be seen on the X‐ray, hence improving the lifestyle of the patient [4]. Nevertheless, methotrexate being an antimetabolic agent may cause adverse effects on the bone marrow, such as cytopenia; other complications include serious infections, liver damage, mucocutaneous problems and hypersensitivity pneumonitis [5]. Here, we document a middle-aged male from Pakistan who has been on methotrexate therapy for the past one year and now presents with painful swallowing, hoarseness of voice, drooling and fresh rectal bleeding.

## Case presentation

A 50-year-old male, who was diagnosed with rheumatoid arthritis one year ago, presented with symptoms involving the small joints of fingers of both hands, symmetrically. As described by the patient, the joints of the fingers were painful and rigid on movement. Furthermore, they appeared swollen on examination. Since then, the patient has been on an active regimen of disease-modifying anti-rheumatic agents ever since. The preferred DMARD for our patient was chosen as methotrexate from the start because the patient was relatively healthy with no previous history of any contraindication of MTX; similarly, it is an easy drug to come around in Pakistan along with its high effectivity in treating the symptoms. He admitted to the use of diclofenac for treating the symptoms of joint pain; no other therapy was used before the diagnosis. Baseline levels before the initiation of therapy were done to compare the before and after functionality of the bone marrow, liver and kidneys which are as follows (Tables 1-3).

**Table 1:** Baseline CBC, CRP TLC: total leukocyte count; CBC: complete blood count; CRP: C-reactive protein.

Parameter	Values	Reference
TLC	10.11	4.5-11x10^9/L
Hemoglobin	15.6	14-16.5 g/dl
RBC	5.0	4.45x10^12/L
Platelets	340	150-400x10^9/L
CRP	4.5	Less than 5.0 mg/L

**Table 2:** Baseline LFTs LFT: Liver Function Test, INR: international normalized ratio.

Parameter	Values	Reference
Bilirubin	1.09	0.1-1.0 mg/dl
Alanine transaminase (ALT)	45.60	10-50 U/L
Alkaline phosphatase (ALP)	101	40-129 U/L
Prothrombin time	1.4	INR 0.9-1.1 seconds

**Table 3:** Baseline RFTs RFT: Renal Function Test.

Parameter	Values	Reference
Blood urea	34.5	10-50 mg/dL
Osmolarity	300	280-295 mmol/L
Creatinine	0.8	0.64-1.2 mg/dL
Creatinine clearance	80	97-137 ml/min
Sodium	150	135-150 mmol/L
Potassium	3.2	3.5-5.1 mmol/L
Chloride	106	96-112 mmol/L

As is the process for any therapy, the patient was started on a low dose of methotrexate initially (10 mg per week). In some cases, the doctors are forced to increase the dosage of the drug to 25 mg per week if the low dose is not helping, but, in our case, the patient reported well improvement in symptoms with this dosage.

Due to the points mentioned above and as the patient was comparatively young, he had no frequent follow-ups. Furthermore, on some occasions that he did visit the doctor, he had no complaints in regard to the treatment; hence, his dosage was kept constant throughout the duration of therapy. Then after being on low-dose methotrexate therapy for almost one year, the patient developed a subacute history of dysphagia or odynophagia, hoarseness of voice and drooling. The difficulty in swallowing, as described by the patient, was slow in onset with a characteristic feeling of food sticking in the throat, accompanied by pain. It was more for solids than for liquids. It had progressed over time, associated with hoarseness in his voice. The patient also complained of fresh rectal bleeding. It was mild and not associated with pain. Other associated symptoms included high-grade fever and productive cough.

Examination of the oral mucosa and rectum showed mucositis as there was difficulty and pain in swallowing, edema and ulcers in the mouth and larynx, leading to the hoarseness in his voice. Ulcers were also found around the rectum accompanied by fourth-degree hemorrhoids. Besides this, the abdomen was soft and non-tender. The cranial nerves were intact. Furthermore, auscultation of the chest revealed bilateral crepts in the lower zone. The rest of the systemic examination was unremarkable.

Prompt action was taken to stabilize the patient with adequate analgesia, fluid replacement and antibiotic cover as a supportive therapy; for this purpose, 1 ml dexamethasone injection, 1 l normal saline infusion and injectable antibiotics, in a combination of piperacillin and tazobactam 4.5 g, were started twice daily. The blood loss was mild so it was sufficed with fluid therapy; hence, no blood transfusion was required. For further workup and investigations, the patient was shifted to the ward. Blood samples were collected, and an ultrasound of the neck and abdomen was carried out. The investigations conducted on days 1, 2 and 3 are listed in Table 4.

**Table 4:** Laboratory values TLC: total leukocyte count, CRP: C-reactive protein, TSH: thyroid-stimulating hormone, ACTH: adrenocorticotropic hormone, INR: international normalized ratio.

Parameter	Day 1	Day 2	Day 3	Reference
TLC	0.44	0.33	0.37	4.5-11x10^9/L
Hemoglobin	7	9.1	10.5	14-16.5 g/dl
Platelets	73	56	31	150-400x10^9/L
CRP	275.26	281	279	Less than 5.0 mg/L
Bilirubin	0.19	1.15	2.0	0.1-1.0 mg/dl
Alanine transaminase (ALT)	19.7	20.3	17.4	10-50 U/L
Alkaline phosphatase (ALP)	67	56	80	40-129 U/L
Prothrombin time	1.4	1.6	1.2	INR 0.9-1.1 seconds
Blood urea	48.3	56.6	38.7	10-50 mg/dL
Creatinine	1	1.37	0.97	0.64-1.2 mg/dL
Sodium	143	126	139	135-150 mmol/L
Potassium	4	5.5	4.7	3.5-5.1 mmol/L
Chloride	116	109	120	96-112 mmol/L
TSH	4.1	2.9	3.6	0.4-5 µIU/ml
Total triiodothyronine (T3)	167	137	159	1.2-2.8 ng/dL
Total thyroxine (T4)	5.6	11.3	9.9	5.4-11.5 µg/dL
ACTH	46.3	62.4	59.3	5.0-60 pg/mL

According to Table 4, the complete blood count showed pancytopenia as hemoglobin was 7, total leukocyte count was 0.4 and the platelet count was 73,000 on the first day. Furthermore, the peripheral smear showed hypocellularity with pancytopenia. Another significant finding was a C-reactive protein value of 275. Urea and electrolytes, serum electrolytes and liver function tests were normal. Virology, malarial parasite and dengue (antigen/serology) were also negative. Other than this, ultrasound abdomen and neck were also reported as normal.

Due to suspected DMARD-associated mucositis, methotrexate was withheld for the time being. Also, leflunomide being similar in profile to MTX and with the suspicion of it being acting in contribution with MTX as the cause of the patient’s symptoms was also stopped. Pharmacological treatment with steroids and an ipratropium bromide nebulizer was started. He was given nystatin drops orally for the symptoms of mucositis in a dose of 1 ml, six hourly. The patient was also commenced on filgrastim (a colony-stimulating factor) 150 mg, twice daily, for three days and injectable folinic acid (leucovorin 15 mg, twice daily) for specific treatment of pancytopenia.

A bone marrow biopsy was done as a part of further workup. It showed cellular fragments that were mixed with areas of hypocellularity; along with this, erythropoiesis and megakaryocytosis were present and normal. The main finding was the presence of all stages of myelopoiesis, dysplastic changes like giant myelocytes, hypogranular myelocytes and karyorrhexis. These findings led to the mere exclusion of aplastic anemia as a differential and were highly suggestive of hypoplastic myelodysplasia. Besides this, multi-parameter flow cytometry for myelodysplastic immunophenotypic aberrancies was done which had non-significant results. After coming across these bone marrow findings and careful review of the history of this patient, consultation with his primary hematologist led to the diagnosis of methotrexate-induced hypoplastic myelodysplasia. The patient was observed on this treatment and followed up in two-week time. Cellular counts which were done during the follow-up appointment showed improvement prompting the reintroduction of low-dose methotrexate for the treatment of rheumatoid arthritis.

## Discussion

Patients with rheumatoid arthritis are liable to the chronic side effects of methotrexate because of the reason that if methotrexate is effective, patients usually receive several years of treatment without measures of long-term safety being acknowledged [5]. According to Pappas et al., among all the patients being treated with MTX for RA, approximately 37% will discontinue the MTX therapy due to the development of complications and other reasons [6]. Among these, pancytopenia is found to be one of the most lethal complications of low-dose MTX therapy [7].

Methotrexate is an antifolate drug; hence, DNA synthesis in all cell lines is hampered. Acquired cases of aplastic anemia (AA) can cause a major hindrance in the diagnosis of hypoplastic myelodysplastic syndrome (hMDS) because of the fact that both are hematopoietic stem cell disorders characterized by pancytopenia with hypocellular bone marrow. Both of these diseases share similar clinical features such as symptoms of fatigue, frequent infections, unexplained or easy bruising, nosebleeds, bleeding gums or any bleeding that lasts longer than expected and pallor, hence making the diagnosis difficult. However, careful history taking, bone marrow analysis showing dysgranulopoiesis, dysmegakaryocytopoiesis, an increased percentage of blasts (which are not found in AA) and abnormal karyotype drug monitoring in patients taking low-dose MTX can help establish the correct diagnosis of hypoplastic myelodysplastic syndrome related to the MTX therapy [8-9].

There are no specific guidelines for effectively treating MTX-induced pancytopenia. However, the treatment strategies that are being used are primarily directed at the reversal of the methotrexate-related toxic effects and its maintenance by continuous monitoring of the plasma concentrations of the drug [10]. This can be achieved by abrupt withdrawal of methotrexate and simultaneously adding anti-folic acid antagonist and synthetic colony-stimulating factors.

Abrupt withdrawal of methotrexate helps to alleviate and limit the adverse effects, which is also reported by Kawase et al. [9]. Leucovorin is an antidote for anti-folic acid agents, as it plays a significant role in reversing MTX-induced myelosuppression. Likewise, a review by Dhillon et al. [10] found that adding filgrastim (a colony-stimulating factor) improves cell count in febrile neutropenia. The dosage regimen was as follows: folinic acid as 15 mg dose I/V, 6 hourly and filgrastim as 150 mg S/C, 12 hourly.

The management of methotrexate-induced hypoplastic myelodysplasia is dependent on the early identification of the symptoms and early initiation of effective treatment. Major issues involve the need to educate the caregivers on careful monitoring of methotrexate as it is often ignored in patients with rheumatoid arthritis; this is, in part, because of the drug being given in low doses as compared to other diseases [11].

## Conclusions

In conclusion, patients on prolonged methotrexate therapy can present with certain symptoms of toxicity related to the liver, bone marrow and gastrointestinal tract. The dosage being lower as compared to other diseases, the patients with rheumatoid arthritis are therefore not kept under constant supervision which may lead to severe complications. Hypoplastic myelodysplastic syndrome is a rare complication of low-dose methotrexate therapy, which was found positive in our patient. The documentation of cases like these, in which low-dose methotrexate leads specifically to myelodysplasia, is rather limited due to which we were unable to compare and contrast the reasons leading to this rare but lethal complication.
